# Young adult breast cancer patients have a poor prognosis independent of prognostic clinicopathological factors: a study from the Japanese Breast Cancer Registry

**DOI:** 10.1007/s10549-016-3984-8

**Published:** 2016-09-19

**Authors:** Akemi Kataoka, Takayuki Iwamoto, Eriko Tokunaga, Ai Tomotaki, Hiraku Kumamaru, Hiroaki Miyata, Naoki Niikura, Masaaki Kawai, Keisei Anan, Naoki Hayashi, Shinobu Masuda, Koichiro Tsugawa, Kenjiro Aogi, Takanori Ishida, Hideji Masuoka, Kotaro Iijima, Takayuki Kinoshita, Seigo Nakamura, Yutaka Tokuda

**Affiliations:** 1 Breast Surgical Oncology Department, Cancer Institute Hospital of Japanese Foundation for Cancer Research, Tokyo, Japan; 2 Department of Breast and Endocrine Surgery, Okayama University Hospital, 2-5-1 Shikata-cho, Kitaku, Okayama City, Okayama 700-8558 Japan; 3 Department of Breast Oncology, National Hospital Organization Kyushu Cancer Center, Fukuoka, Japan; 4 Department of Healthcare Quality Assessment, Graduate School of Medicine, Tokyo University, Tokyo, Japan; 5 Department of Breast and Endocrine Surgery, Tokai University School of Medicine, Kanagawa, Japan; 6 Department of Breast Surgery, Miyagi Cancer Center, Natori, Japan; 7 Department of Surgery, Kitakyushu Municipal Medical Center, Kitakyushu, Japan; 8 Department of Breast Surgery, St. Luke’s International Hospital, Tokyo, Japan; 9 Department of Pathology, Nihon University School of Medicine, Tokyo, Japan; 10 Division of Breast and Endocrine Surgery, Department of Surgery, St. Marianna University School of Medicine, Kawasaki, Japan; 11 Department of Breast Surgery, Shikoku Cancer Center, Matsuyama, Japan; 12 Department of Surgical Oncology, Graduate School of Medicine, Tohoku University, Sendai, Japan; 13 Sapporo-kotoni Breast Clinic, Sapporo, Japan; 14 Department of Breast Oncology, Juntendo University School of Medicine, Tokyo, Japan; 15 Department of Breast Surgery, National Cancer Center Hospital, Tokyo, Japan; 16 Division of Breast Surgical Oncology, Department of Surgery, Showa University, Tokyo, Japan

**Keywords:** Breast cancer, Young women, Surveillance data, Prognosis, Multivariable analysis

## Abstract

**Purpose:**

The aim of this study was to investigate whether young age at onset of breast cancer is an independent prognostic factor in patients from the Japanese Breast Cancer Registry, after adjustment of known clinicopathological prognostic factors.

**Methods:**

Of the 53,670 patients registered between 2004 and 2006 and surveyed after a 5-year follow-up prognosis, 25,898 breast cancer patients (48.3 %), who were obtained prognostic data, were examined. Clinicopathological factors were compared between young adult (YA; <35 years), middle-aged adult (MA; 35–50 years), and older adult (OA; >50 years) patients. Five-year disease-*free* survival (DFS) and overall survival (OS) rates were studied.

**Results:**

YA patients were associated with an advanced TNM stage and aggressive characteristics (e.g. human epidermal growth factor receptor 2 (HER2)-positive or oestrogen receptor (ER)-negative breast cancers) compared to MA and OA patients (*P* < 0.001). The 5-year DFS and OS rates were 79.4 % and 90.8, 88.5 and 95.0 %, and 87.8 % and 91.6 % for YA, MA, and OA patients, respectively. From the multivariable regression analysis, young age at onset was confirmed as an independent prognostic factor for both DFS (hazard ratio 1.73, 95 % confidence interval 1.42–2.10; *P* < 0.001) and OS (hazard ratio 1.58, 95 % confidence interval 1.16–2.15; *P* = 0.004).

**Conclusions:**

Young age at onset is an independent negative prognostic factor in breast cancer. Further studies are required to develop new therapeutic strategies for YA breast cancer patients.

**Electronic supplementary material:**

The online version of this article (doi:10.1007/s10549-016-3984-8) contains supplementary material, which is available to authorized users.

## Introduction

Young adult (YA) cancers are relatively rare and represent a minority of cases. Consequently, data are lacking concerning intellectual and other psychosocial issues affecting this specific patient population [1]. YA cancer patients are significantly more likely to indicate unmet needs for supportive care services [2]. Moreover, fewer clinical trials have been conducted for YA cancers compared to other adult cancers, suggesting that there may be little evidence of high impact. Among YA cancers in women, breast cancer has the highest incidence rates (30–34 years, 13.3 per 100,000 population, and 35–39 years, 31.6 per 100,000 population [3]. However, even breast cancers account for a very small proportion (approximately 7 %) of the total number of breast cancers in these age groups [4–6].

YA breast cancer patients diagnosed in their twenties or thirties tend to have a poorer prognosis than women diagnosed in middle age (MA) [7]. Differences in survival may reflect clinical and biological variations. Indeed, YA breast cancer patients are reported to present with more aggressive biological characteristics and to behave more poorly compared to older breast cancer patients [8]. Previously, we reported the clinicopathological features of YA patients as having advanced TNM staging and human epidermal growth factor receptor 2 (HER2)-positive/oestrogen receptor (ER)-negative breast cancers compared to older patients [6]. Similarly, aggressive and unfavourable characteristics, including TNM classification, ER status, and HER2 status for YA patients with breast cancer have been reported [9–13].

However, to our knowledge, most of the data on the biological characteristics and treatment to evaluate these patients were derived from older and relatively smaller cohort studies. Moreover, whether age remains an independent predictive prognostic factor, after adjustment of breast cancer subtype (ER, PR, and HER2 status), as well as, other known prognostic factors (TNM classification, adjuvant systemic therapy, etc.) has yet to be determined, given YA patients are at risk of developing more aggressive and more advanced breast cancers.

The aim of this study was to investigate whether young age at onset of breast cancer is an independent negative prognostic factor in patients from the Japanese Breast Cancer Registry (which includes >25,000 newly treated breast cancers between 2004 and 2006).

## Materials and methods

### Patients

This study was conducted using the Japanese Breast Cancer Registry database, the details of which have been reported previously by Kurebayashi et al. [14]. Briefly, it is a registry managed by the Registration Committee of the Japanese Breast Cancer Society with support from the Public Health Research Foundation (Tokyo, Japan). Data on newly operated primary breast cancer patients are reported from affiliated institutes throughout Japan, which included 741 facilities in 2011, through a web-based system that collects information on >50 demographic and clinicopathological characteristics. Pathological TNM classification is registered based on the Unio Internationalis Contra Cancrum staging system (sixth edition) [15]. Histological classification is registered according to the General Rules for Clinical and Pathological Recording of Breast Cancer [16], which has been translated into the Classification of Tumours of the Breast and Female Genital Organs [17]. Age at onset was defined as the age of the beginning of treatment.

**Fig. 1:**
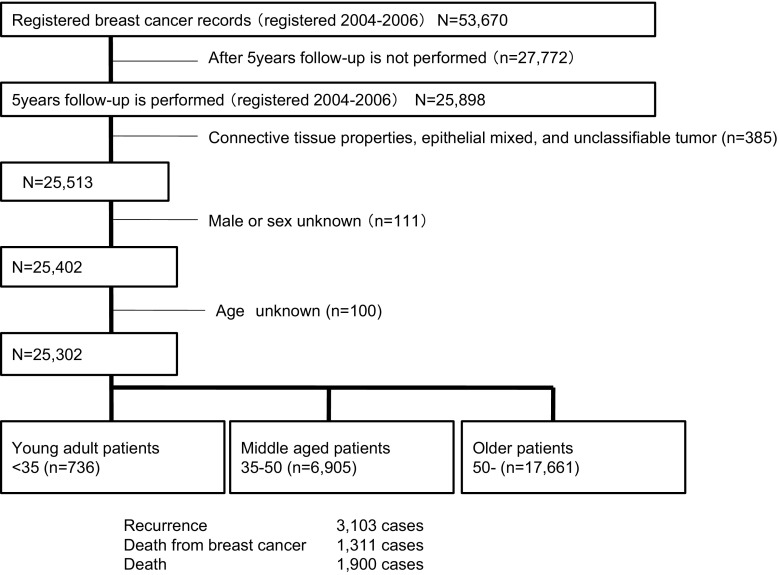
Patient flow chart

HER2 positivity was defined as immunohistochemical staining of 3+ or a positive fluorescent in situ hybridisation test according to the manufacturer’s criteria. Hormone receptor (ER/progesterone receptor [PR]) positivity was determined if ≥1 % of nuclei in the tumour stained positive for ER/PR on immunohistochemical analysis. Of the 53,670 patients registered in the Japanese Breast Cancer Registry between 2004 and 2006 and surveyed after a 5-year follow-up prognosis, 25,898 patients (48.3 %) were obtained follow-up data and used for further examinations. Cases with connective tissue properties and mixed epithelial or unclassified tumours (*n* = 385) were excluded, as were male cases and cases of unknown age or sex (*n* = 211). A patient flow chart is depicted in Fig. 1. In total, 25,302 patients were analysed in this study. YA breast cancer patients (*n* = 736; 2.9 %) were defined as <35 years of age, MA patients (*n* = 6905; 27.3 %) as between 35 and 50 years of age at onset, and OA patients (*n* = 17,661; 69.8 %) as >50 years of age at onset. Clinicopathological and prognostic factors were compared between the three groups. For the analysis of survival, patients who did not undergo surgery (*n* = 312; 1.2 %), patients who had Stage IV or an unknown disease stage (*n* = 987; 3.9 %), and patients with unavailable event data (*n* = 212; 0.8 %) were excluded.

### Statistical analyses

Fisher’s exact tests were used to compare various prevalence rates among the three patient groups. Unpaired Student’s *t*-tests were used for inter-group comparisons of continuous variables. Survival curves were constructed using the Kaplan–Meier method with and without stratification on known prognostic factors, and were compared using a log-rank test. Multivariable analyses for disease-*free* survival (DFS), breast cancer-specific survival (BCSS), and overall survival (OS) were performed using a Cox proportional hazards model to estimate the hazard ratios and 95 % confidence intervals for survival. We considered the following variables as potential confounders in the Cox model; age, TNM classification, breast cancer subtype, and neo-adjuvant/adjuvant therapy. Patients with any missing or unknown data were excluded from analysis of the Cox model. DFS was defined as the time interval between the date of surgery and the point of local or distant recurrence. BCSS and OS were defined as the time intervals between the date of surgery and the date of breast cancer-related death or death from any cause. A *P* value of <0.05 was considered statistically significant. All statistical analyses were conducted using SAS software version 9.4 (SAS Institute, Inc., Cary, NC, USA).

## Results

### Clinicopathological characteristics

Prognostic information was available for 736 YA patients (2.9 %), 6905 MA patients (27.3 %), and 17,661 OA patients (69.8 %), indicating that the minority of all breast cancers are YA cases, as previously reported (Table 1) [4–6].

**Table 1:** Patient characteristics^a^

	Patients’ age at onset						
	<35 (*n* = 736)		35–50 (*n* = 6905)		50– (*n* = 17,661)		
	*N*	%	*N*	%	*N*	%	*P* value
T stage							
Tis	73	9.9	795	11.5	1580	9.0	**<.001**
T0	5	0.7	97	1.4	233	1.3	
T1	229	31.1	2655	38.5	6870	38.9	
T2	301	40.9	2434	35.3	6475	36.7	
T3	93	12.6	579	8.4	1243	7.0	
T4	26	3.5	280	4.1	1109	6.3	
Unk	9	1.2	65	0.9	151	0.9	
Nodal status							
Negative	515	70.0	5281	76.5	13,625	77.2	**<.001**
Positive	210	28.5	1547	22.4	3825	21.7	
Unk	11	1.5	77	1.1	211	1.2	
M							
M0	692	94.0	6640	96.2	16848	95.4	**<.001**
M1	29	3.9	128	1.9	461	2.6	
Unknown	15	2.0	137	2.0	352	2.0	
Stage							
0	73	9.9	795	11.5	1576	8.9	**<.001**
1	202	27.5	2468	35.7	6354	36.0	
2	338	45.9	2886	41.8	7499	42.5	
3	78	10.6	535	7.8	1511	8.6	
4	29	3.9	128	1.9	461	2.6	
Unknown	16	2.2	93	1.4	260	1.5	
ER							
Negative	195	26.5	1307	18.9	4578	25.9	**<.001**
Positive	517	70.2	5353	77.5	12544	71.0	
Unknown	24	3.3	245	3.6	539	3.1	
PR							
Negative	263	335.7	1647	23.9	7594	43.0	**<.001**
Positive	447	60.7	4997	72.4	9460	53.6	
Unknown	26	3.5	261	3.8	607	3.4	
HER2							
Negative	554	75.3	5231	75.8	12961	73.4	**<.001**
Positive	101	13.7	806	11.7	2582	14.6	
Unknown	81	11.0	868	12.9	2118	12.0	
Surgery							
None	1	0.1	4	0.1	18	0.1	**<.001**
BCT	456	62.0	4070	58.9	9092	51.5	
Mastectomy	256	34.8	2671	38.7	8115	45.9	
Others	16	2.2	97	1.4	217	1.2	
Unknown	7	1.0	63	0.9	219	1.2	
Adjuvant therapy							
None	126	17.1	1036	15.0	3414	19.3	**<.001**
ET	252	34.2	2899	42.0	7725	43.7	
ET + CT	197	26.8	1834	26.6	3133	17.7	
CT	122	16.6	879	12.7	2701	15.3	
Unknown	39	5.3	257	3.7	688	3.9	

Bold *P* value <0.05

^a^TNM classification is shown based on the sixth edition of the Unio Internationalis Contra Cancrum staging system; *ER* estrogen receptor, *PR* progesteron receptor, *HER2* human epidermal growth factorreceptor 2, *BCT* breast conserving therapy, *ET* endocrine therapy, *CT* Chemo therapy

YA patients were more likely to be diagnosed with a larger tumour (e.g., T3: YA patients, 12.6 %; MA patients, 8.4 %; and OA patients, 7.0 %; *P* < 0.001), Tis (ductal carcinoma in situ) occurred most frequently in MA patients (11.5 %) and T1 occurred more frequently in MA (38.5 %) and OA patients (38.9 %) compared to YA patients (31.1 %; *P* < 0.001). A greater proportion of YA patients (28.5 %) had a positive nodal status compared to MA (22.4 %) and OA patients (21.7 %; *P* < 0.001). Distant metastasis (M status) also occurred significantly more frequently in YA patients compared to MA and OA patients (*P* < 0.001). Moreover, an advanced TNM classification (Stage III/IV) occurred more frequently in YA patients (14.5 %) compared to MA (9.6 %) and OA patients (11.2 %; *P* < 0.001). YA patients were also associated with an aggressive breast cancer receptor status. Specifically, the proportion of ER-negative tumours was higher in YA patients compared to MA and OA patients (*P* < 0.001), although the difference in frequencies between YA (26.5 %) and OA patients (25.9 %) was small. A similar trend was observed in the HER2-positive group in which YA patients (13.7 %) were more frequent than MA patients (11.7 %) (*P* < 0.001; Table 1).

In regard to the type of surgery conducted, YA patients (62.0 %) underwent BCT more frequently compared to MA (58.9 %) and OA patients (51.5 %; *P* < 0.001). Adjuvant systemic therapies (endocrine therapy alone, combination chemo-endocrine therapy, chemotherapy alone, and no adjuvant therapy) were also compared. The uptake of adjuvant endocrine therapy alone was significantly lower in YA patients compared to MA and OA patients (*P* < 0.001). Conversely, YA patients were administered chemotherapy and combination chemo-endocrine therapy more frequently compared to MA and OA patients (*P* < 0.001; Table 1).

### Prognosis

At 5-year follow-up, 3103 cases (12.3 %) of breast cancer recurrence, 1311 cases (5.2 %) of breast cancer-related death, and 1900 cases (7.5 %) of all-cause death were reported. The 5-year DFS rates were 79.4, 88.5, and 87.8 % for YA, MA, and OA patients, respectively. The 5-year BCSS and OS rates were 92.1 and 90.8 % for YA, 95.8 and 95.0 % for MA, and 94.6 and 91.6 % for OA patients.

**Fig. 2:**
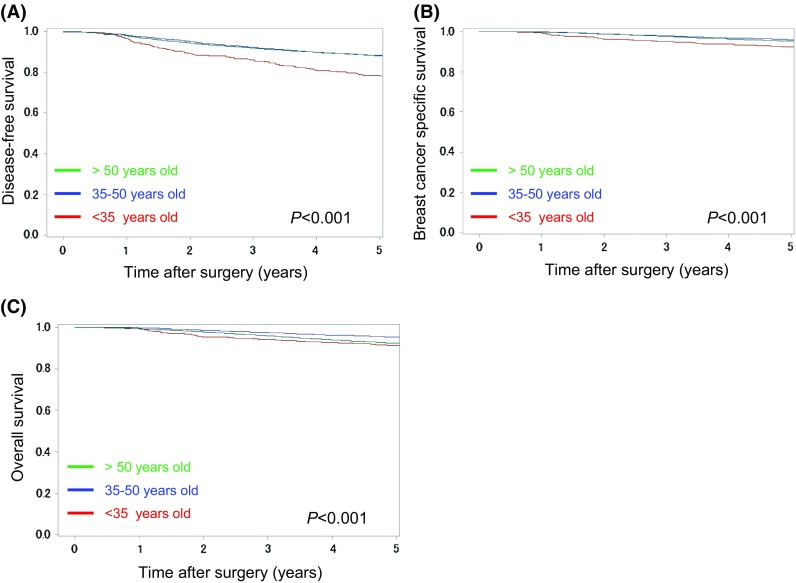
Kaplan–Meier curves for **a** disease-*free* survival, **b** breast cancer-specific survival, and **c** overall survival between young adult (<35 years; *red line*), middle-aged adult (35–50 years; *blue line*), and older adult (>50 years; *green line*) breast cancer patients. *P*-values were calculated using a log-rank test

**Fig. 3:**
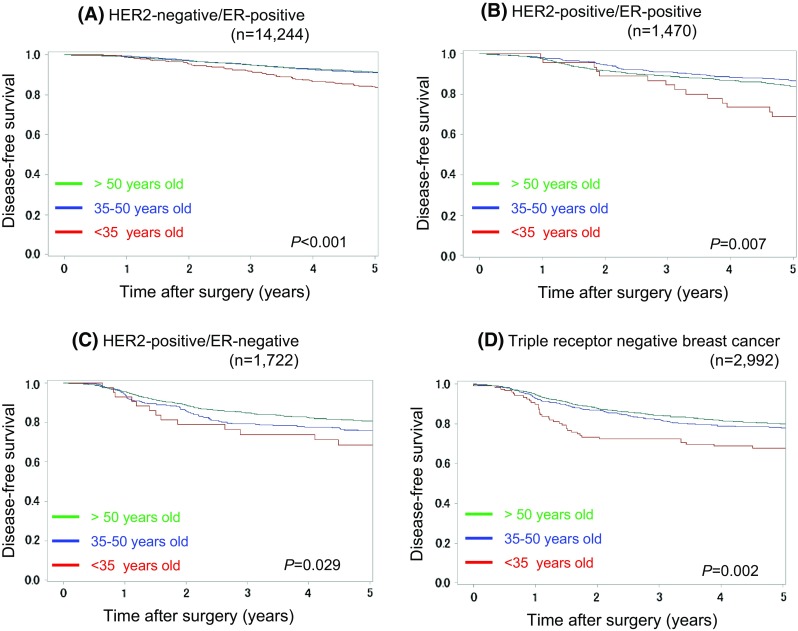
Kaplan–Meier curves for disease-*free* survival between young adult (<35 years; *red line*), middle-aged adult (35–50 years; *blue line*), and older adult (>50 years; green line) patients with **a** HER2-negative/ER-positive, **b** HER2-positive/ER-positive, **c** HER2-positive/ER-negative, and **d** triple receptor negative breast cancer. *P*-values were calculated using a log-rank test

YA patients were associated with a significantly poorer prognosis in relation to DFS, BCSS, and OS (*P* < 0.001; Fig. 2) in the univariate analysis, indicating that these results are consistent with previously reported data [9–13]. We subsequently assessed the prognostic value of young age at onset in breast cancer, stratifying on known clinicopathological prognostic factors. Stratifying on breast cancer receptor status (HER2-positive/ER-positive, HER2-positive/ER-negative, HER2-negative/ER-positive, and triple receptor negative breast cancer), YA patients were found to be significantly associated with a poorer prognosis in all breast cancer receptor subtypes (*P* < 0.05; Fig. 3). In ER-positive cases, there was no difference on recurrence pattern by age at onset in the early phase during this study period, and YA cases had poorer prognosis than the older cases in the late phase(Fig. 3a, b). Conversely, in ER-negative cases, the distinct pattern of the recurrence by age at onset was seen only in the early phase and no difference in the late phase(Fig. 3c, d). TNM stage, another well-known clinicopathological prognostic factor, was also stratified. YA patients were associated with a significantly poorer prognosis in the Stage I and Stage II groups (*P* < 0.001; Fig. S1). In the Stage 0 group, YA, MA, and OA patients with ductal carcinoma in situ were associated with similarly favourable prognoses with statistically marginal effect (*P* = 0.053; Fig. S1). Conversely, in the Stage III group, YA patients exhibited a trend towards a poorer prognosis. However, this was not statistically significant (*P* = 0.121; Fig. S1).

Finally, multivariable Cox regression analysis was performed using a young age at onset adjusted by known breast cancer prognostic factors, including T/N status, breast cancer subtypes, and adjuvant therapies. YA patients were significantly associated with the poorest prognosis for all three endpoints, 5-year DFS, BCSS, and OS. Specifically, both comparisons between YA and MA patients (hazard ratio 1.58, 95 % confidence interval 1.16–2.15; *P* < 0.01) and between YA and OA patients (hazard ratio: 1.52, 95 % confidence interval 1.33–1.75; *P* < 0.001) were significant for OS (Table 2).

**Table 2:** Multivariate analysis for 5 year survival^a^

	Hazard ratio	95 % C.I.	*P value*	Hazard ratio	95 % C.I.	*P value*	Hazard ratio	95 % C.I.	*P value*
Age at diagonosis									
<35 versus 35–50	1.73	1.42–2.10	**<.001**	1.52	1.09–2.13	0.098	1.58	1.16–2.15	**0.004**
Over 50 versus 35–50	0.99		0.821	1.14	0.98–1.34	**0.015**	1.52	1.33–1.75	**<.001**
T									
T2–4 versus T0, 1	2.22	0.90–1.09	**<.001**	3.04	2.49–3.70	**<.001**	2.25	1.96–2.59	**<.001**
N									
Positive versus negative	2.81	2.01–2.46	**<.001**	4.01	3.46–4.64	**<.001**	3.05	2.72–3.43	**<.001**
Breast cancer subtype									
ER+HER2+ versus ER+HER2−	1.52	2.58–3.07	**<.001**	1.73	1.35–2.23	**<.001**	1.39	1.13–1.72	**0.002**
ER−HER2+ versus ER+HER2−	1.86	1.65–2.11	**<.001**	2.33	1.89–2.88	**<.001**	1.75	1.47–2.07	**<.001**
Triple negative versus ER + HER2−	2.06	1.86–2.28	**<.001**	4.48	3.84–5.23	**<.001**	3.08	2.72–3.50	**<.001**
Adjuvant therapy									
Any versus none	0.87	0.76–1.00	**0.041**	1.32	1.03–1.71	**0.032**	0.70	0.60–0.81	**<.001**

Bold P value < 0.05

^a^ *DFS* disease-*free* survival, *BCSS* breast cancer specific survival, *OS* overall survival; TNM classification is shown based on the 6th edition of the Unio Internationalis Contra Cancrum staging system; *ER* estrogen receptor, *HER2* human epidermal growth factor receptor 2

## Discussion

YA breast cancer accounts for a minority of breast cancer cases [6].

Consequently, it is unlikely that a prospective clinical trial would ever be conducted to define the optimal treatment strategy for this disease subset.

We analysed data from a large number of breast cancer patients registered by the Japanese Breast Cancer Registry database in order to characterise and advance our understanding of YA breast cancer. Using nationwide, population-based data representing approximately 70 % of all newly diagnosed breast cancer patients in Japan between 2004 and 2006, we were able to circumvent many problems associated with single institutional experiences or limited sample sizes. Our study demonstrated that a young age at onset was an independent predictive factor for poor prognosis in patients with breast cancer, after adjustment of well-known clinicopathological factors, including breast cancer receptor status, tumour size, and nodal status. Classically, it has been suggested that YA breast cancer patients are associated with a poorer prognosis because of delayed diagnosis at an advanced stage, a larger tumour size, and higher incidences of HER2-positive/ER-negative tumours [6, 9]. These reports proved consistent with our findings in the present study. Some previously published studies have already established a poorer prognosis in YA breast cancer patients as independent from other clinicopathological factors, such as tumour size, nodal status, histological grade, and hormone receptor status [8, 18, 19]. However, these reports are relatively old, have smaller sample sizes, and patients may have been treated with a classical adjuvant chemotherapy and endocrine therapy regimen. Recently, some studies using large databases have also reported similarly poor prognostic outcomes in YA breast cancer patients after stratifying on multiple prognostic factors [20–23]. Conversely, a single study has found that a young age at onset has no influence on the prognosis of individual breast cancer patients from a database of almost 3000 cases [24]. Partridge et al. [12] also reported no effect of age on breast cancer outcomes in patients with HER2-positive breast cancer from a large, randomised controlled trial. At the St Gallen International Expert Consensus meetings, a younger age at onset had been considered a high-risk factor from the 1990s to 2009. Later, a younger age at onset was no longer considered to be a poor prognostic factor and treatment strategies were recommended based on biological subtype or the concept of a ‘threshold for indication’ of each systemic treatment modality to be respected without a young age at onset [25]. Then, YA patients were treated according to various predictive factors and the subtype of the tumour, including ER, PR, and HER2 status, proliferation markers, and TNM classification and a young age itself had no impact on the treatment strategy. Based on our findings and the results of several previously published reports of large cohorts [20–23], YA breast cancer patients have a poor prognosis independent of other aggressive breast cancer features.

Another interesting finding was distinct recurrence pattern between ER-positive and -negative entities according to age at onset (Fig. 3). These differences between age at onset and ER status may lead to the distinct biological and molecular processes of age at onset by ER status. Research highlighting the genetic differences between YA and other breast cancer entities by ER status is lacking. Anders et al. [11] reported that YA breast cancer represents a unique biological entity driven by unifying a higher probability of phosphoinositide 3-kinase and Myc pathway dysregulation. Investigating how high-risk genetic mutations affect age at onset, Ford et al. [26] observed that 5.3 % of breast cancers in <40 year olds are attributable to *BRCA1* mutations compared 2.2 % and 1.1 % in 40- to 49-year olds and 50- to 70-year olds, respectively. It has been established that patients with *BRCA1* mutations are more likely to develop basal-like breast cancers, including the triple-negative subtype [27, 28] [29, 30]. Further research to elucidate the development of disease in this high-risk YA population and to determine the prognosis following a diagnosis of breast cancer is clearly warranted. An improved understanding of breast cancer genetics through molecular profiling may provide information that can be applied to patients with YA breast cancer.

Efficacy to adjuvant therapy in YA breast cancer patients remains controversial. Ahn et al. [10] reported that the survival differences according to age in hormone receptor-positive breast cancer patients were significant in patients who received hormone therapy as well as those who did not. This suggests YA breast cancer patients may need another strategy of treatment instead of conventional adjuvant hormone and chemo therapy. A similarly insufficient efficacy to chemotherapy has also been reported. YA breast cancer patients treated with adjuvant cyclophosphamide, methotrexate, and fluorouracil are at a higher risk of relapse and death compared to older breast cancer patients [31].

These distinct genetic patterns and clinical outcomes may lead to individual management of breast cancer patients. Previous studies reported significantly higher rates of local recurrence in YA patients who received BCT compared to OA patients who underwent a mastectomy [32, 33]. Freedoman et al. [34] reported that YA breast cancer patients were significantly more likely to have a mastectomy than BCT compared to older breast cancer patients. Efforts are required to confirm whether different types of surgery effect not only local recurrence rates but also OS rates. [35].

This study had several limitations. First, the relatively short follow-up period (median 4.5 years), which limited the power of the survival analysis. Nevertheless, prognostic analyses from this database that have previously been published were relatively consistent with the well-known consensus and clinical outcomes [36–38]. Second, during the study period, trastuzumab (which should exert a favourable effect on HER2-positive breast cancers) had not been widely prescribed as the standard agent and was only partially received. Third, we have no proliferation data, such as grade and genomic signatures. They are primarily prognostic and secondary predictive markers to chemotherapy response especially in ER-positive cases.

In conclusion, the present study confirmed that YA breast cancer patients have a poor prognosis independent of well-known clinicopathological prognostic factors. The different prognoses between YA, MA, and OA patients may require different screening algorithms, therapies, and follow-up. In order to establish an optimal strategy for YA breast cancer patients, further studies will need to be conducted.

## Electronic supplementary material

Below is the link to the electronic supplementary material.

Fig. S1: Kaplan–Meier curves for disease-*free* survival between young adult (<35 years; red line), middle-aged adult (35–50 years; blue line), and older adult (>50 years; green line) patients with (**A**) Stage 0, (**B**) Stage I, (**C**) Stage II, and (**D**) Stage III breast cancer. *P*-values were calculated using a log-rank test (PPTX 119 kb)
