# Supplementary material for: Young adult breast cancer patients have a poor prognosis independent of prognostic clinicopathological factors: a study from the Japanese Breast Cancer Registry
Source: Breast Cancer Res Treat. 2016 Sep 19;160(1):163–72. doi: 10.1007/s10549-016-3984-8 (PMC5050233; doi:10.1007/s10549-016-3984-8)
Supplement: Supplementary file 1 — Fig. S1: Kaplan–Meier curves for disease-free survival between young adult (<35 years; red line), middle-aged adult (35–50 years; blue line), and older adult (>50 years; green line) patients with (A) Stage 0, (B) Stage I, (C) Stage II, and (D) Stage III breast cancer. P-values were calculated using a log-rank test (PPTX 119 kb) [file 10549_2016_3984_MOESM1_ESM.pptx]

## Slide 1
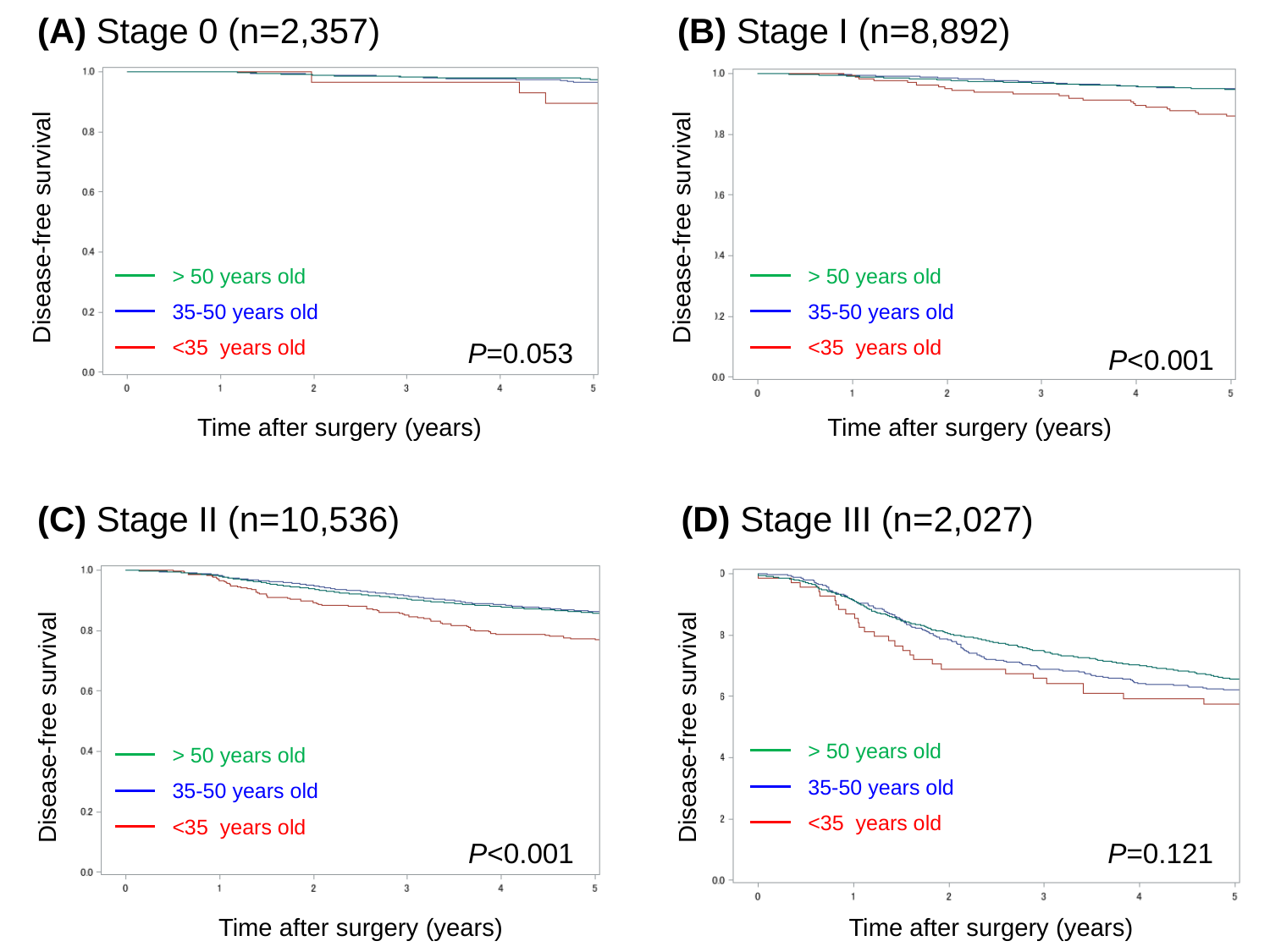

(A) Stage 0 (n=2,357)
(B) Stage I (n=8,892)
Disease-free survival
Disease-free survival
> 50 years old
35-50 years old
<35 years old
> 50 years old
35-50 years old
<35 years old
P=0.053
P<0.001
Time after surgery (years)
Time after surgery (years)
(C) Stage II (n=10,536)
(D) Stage III (n=2,027)
Disease-free survival
Disease-free survival
> 50 years old
35-50 years old
<35 years old
> 50 years old
35-50 years old
<35 years old
P<0.001
P=0.121
Time after surgery (years)
Time after surgery (years)
